# Health Policy and Systems Research in Twelve Eastern Mediterranean Countries: a stocktaking of production and gaps (2000-2008)

**DOI:** 10.1186/1478-4505-9-39

**Published:** 2011-10-07

**Authors:** Fadi El-Jardali, Diana Jamal, Nour Ataya, Maha Jaafar, Saned Raouf, Claudia Matta, Saja Michael, Colette Smith

**Affiliations:** 1 Department of Health Management and Policy, Faculty of Health Sciences, American University of Beirut, Beirut, Riad El Solh 1107 2020, Lebanon

## Abstract

**Background:**

The objectives of this study are to: (1) profile the production of Health Policy and Systems Research (HPSR) published between 2000 and 2008 in 12 countries in the Eastern Mediterranean Region (EMR): Bahrain, Egypt, Jordan, Lebanon, Libya, Morocco, Oman, Palestine, Sudan, Syria, Tunisia, and Yemen; (2) identify gaps; and (3) assess the extent to which existing HPSR produced in the region addresses regional priorities pertaining to Health Financing, Human Resources for Health and the Role of the Non-State Sector. This is the first stocktaking paper of HPSR production and gaps in the EMR.

**Methods:**

Articles indexed on Medline between years 2000 and 2008 for the 12 study countries were selected. A MeSH term based search was conducted using country names. Articles were assessed using a coding sheet adapted for the region which included themes on: Governance Arrangements, Financial Arrangements, Delivery Arrangements, and Implementation Strategies. Identified articles were matched against regional research priorities to assess the extent to which research production aligns with priorities.

**Results:**

A total of 1,487 articles (11.94%) fit the criteria in the coding sheet. Results showed an increase in HPSR production which peaked after 2005. Most identified articles focused on Delivery Arrangements (68.1%), and Implementation Strategies (24.4%). Most HPSR addressed priorities in Human Resources for Health (39%**)**, and some articles focused on Health Financing (12%) and Role of the Non-State Sector (6.1%).

**Conclusions:**

Despite global calls for producing and translating HPSR into policy, there are still significant gaps in the EMR. More efforts are needed to produce HPSR and align production and translation with the demand for evidence by policymakers. Findings can help inform and direct future plans and activities for the Evidence Informed Policy Network- EMR, World Health Organization- EMR, and the Middle East and North Africa Health Policy Forum, in addition to being useful for countries that host or are planning to host KT platforms in the region.

## Background

Evidence has no value except that which can be gained from its application towards some worthy end. Improving health conditions and equitable access to health care is highly reliant on well-informed policies that make use of the best health policy and systems research (HPSR) evidence available. The Alliance for Health Policy and Systems Research (AHPSR) defines HPSR as an emerging field that seeks to understand and improve how societies organize themselves in achieving collective health goals, and how different actors interact in the policy and implementation processes to contribute to policy outcomes [1,2]. It is inter-disciplinary and draws a comprehensive picture of how health systems respond and adapt to health policies, and how health policies can shape - and be shaped by - health systems and the broader determinants of health. HPSR is characterized by the types of questions it addresses. It focuses upon the upstream aspects of health, organizations and policies. It covers a wide range of questions, from financing to governance and implementation of services and delivery of care in both the public and private sectors. It is a crucial policy analysis tool of policies and processes including the role, interests and values of key actors at local, national and global levels [1].

Over the last years, the need for strengthening HPSR and enhancing its translation into policy has been repeatedly emphasized [3-7]. The World Health Assembly Resolution 58.34 urged member states to "establish or strengthen mechanisms to transfer knowledge in support of.... evidence-based health-related policies" [4]. This resolution was later reiterated in the Bamako Call to Action, which urged national governments to allocate at least 2% of budgets of ministries of health to research and 5% of funding for research, including the application of evidence-informed policies, policy-informed research, and the effective dissemination of research results [5].

While the use of research evidence in policymaking is becoming of high priority to many countries at the global level, limited work has been done to date on the generation and utilization of research evidence in the Eastern Mediterranean Region (EMR) [8]. A recent print media analysis in 44 Low and Middle Income Countries (LMICs), which included several countries from the EMR, showed that the region is among the lowest in terms of the articles that describe or use health research evidence [9]. Moreover, there is a deficiency in the production of systematic reviews from the EMR [[8,10], El-Jardali et al. Use of Health Systems Evidence by Policymakers in Eastern Mediterranean Countries: Views, Practices, and Contextual Influences under review, El-Jardali et al. Increasing the Use of Health Systems and Policy Research Evidence in the Health Policymaking in Eastern Mediterranean Countries: Views and Practices of Researchers, under review]. Profiling the production of systematic reviews in the region revealed that only seven of 384 identified reviews addressed health systems topics [10,11]. A study of research output in the EMR showed that health has been de-politicized in much of the published research [12]. Furthermore, the average number of health-related research publications in countries of the EMR of different income groups is far lower than the world average for the same groups, which shows that economic resources are not the only factor responsible for low health research output in the region [13]. Some of the main challenges that hinder the use of evidence in policymaking in the region are; the inadequacy of resources allocated to research, the complexity of the policymaking environment, poor value given to research, difficulty accessing research evidence by researchers and policymakers [[14-16], El-Jardali et al. Use of Health Systems Evidence by Policymakers in Eastern Mediterranean Countries: Views, Practices, and Contextual Influences under review; El-Jardali et al. Increasing the Use of Health Systems and Policy Research Evidence in the Health Policymaking in Eastern Mediterranean Countries: Views and Practices of Researchers, under review; El-Jardali et al. Evidence- to- Policy Advocacy in the Eastern Mediterranean Region (EMR): Assessing the climate for use of evidence and identifying needs for establishing KT platforms. Under review]. In addition, much of the produced research can be found in locally non-indexed journals and as grey literature (i.e. tacit knowledge), and is thus not readily accessible or searchable [17].

Calls for strengthening HPSR and its use in health policymaking in the EMR include the Qatar Declaration, issued in 2008, which urged ministries of health (MOH) and the World Health Organization (WHO) to solicit research that addresses MOH needs and to engage the community and the media to ensure that research addresses community needs and results are communicated to the community. The Qatar declaration called for WHO's support for an in-depth situation analysis of the status of health systems research in the region [18]. More recently, in its strategic directions for research for health, WHO Eastern Mediterranean Regional Office (WHO EMRO) emphasized the forceful implementation and expansion of research for health as a fundamental tool for health development and informing health policy changes [14].

Analysis of HPSR publications in countries from the region can be used to monitor progress and trends in the production of policy- relevant research and is a core requirement for strengthening health research systems to generate and use knowledge to improve health systems [15]. The analysis of research production from the region is mainly focused on biomedical research [17,19-23]; whereas none profiled the production of HPSR. Methods used for conducting stocktaking exercises include the use of validated search strategies to identify systematic reviews in Medline and EMBASE and Web of Science [24-26], media analysis by searching LexisNexis Academic [9,27,28], search strategies to identify public health research in Grey literature [29], as well as surveys of research producers [30].

Recently, a priority-setting exercise with policymakers, stakeholders, and researchers from nine countries in the region identified policy concerns and health systems research priorities related to health financing, human resources for health, and the non- state sector [8]. However, the extent to which existing HPSR that is produced in the region can address these priorities is not yet known. There is a need to map out which of these priorities are already addressed by existing HPSR and which ones require additional primary research or synthesis from existing research [8]. Many countries in the region are currently in the process of developing and expanding national KT platforms aimed at strengthening evidence-informed policymaking. A stocktaking exercise of HPSR production and gaps in the region can complement and inform the planned activities of national KT platforms including priority-setting, systematic reviews, policy briefs, and policy dialogues [El-Jardali et al. Evidence- to- Policy Advocacy in the Eastern Mediterranean Region (EMR): Assessing the climate for use of evidence and identifying needs for establishing KT platforms. Under review].

The objectives of this paper are to: (1) profile the production of HPSR published between 2000 and 2008 in 12 countries in the EMR (Bahrain, Egypt, Jordan, Lebanon, Libya, Morocco, Oman, Palestine, Sudan, Syria, Tunisia, and Yemen); (2) identify gaps in the production; and (3) assess the extent to which the existing HPSR that is produced in the region addresses regional priorities pertaining to Health Financing, Human Resources for Health and the Role of the Non-State Sector as identified in an earlier priority setting exercise. We intend to use the profiles of HPSR production as a baseline assessment against which to compare the future production of HPSR in the region and at the country level and guide future work on strengthening evidence- informed policies in the region.

The countries included in this study were selected through purposive sampling. Specifically, countries were selected based on their interest and participation in the launch meeting of the Evidence Informed Policy Network- Eastern Mediterranean Region (EVIPNet EMR) that took place in January 2009. EVIPNet is a social network that encourages the use of evidence in the policymaking process. It includes researchers, policymakers and civil society members from the EMR.

## Methods

Articles indexed on Medline between year 2000 and 2008 for each of the 12 identified study countries were selected. MEDLINE is the bibliographic database that contains over 18 million references to articles pertaining to life sciences and medicine. Records on MEDLINE are indexed using terms which are known as Medical Subject Headings (MeSH). MEDLINE also includes journals and newsletters related to health services research, history of medicine, AIDS, environmental health and others. The database covers material from the year 1946 and currently cites approximately 5,516 international journals in 39 languages [31].

The search for the articles in this paper was done using MeSH term based on country name. This allowed us to identify all articles published in regional or international journals indexed on MEDLINE. In instances where country names could not be mapped to a MeSH term, keywords were used instead. This was done specifically for Palestine which was searched using three terms: Palestine, West Bank and Gaza and Occupied Palestinian Territories. The search was not limited to full text articles, or English language articles only so as not to reduce or eliminate some articles from the review process. A total of 12,488 unique articles were identified from this search.

We assessed the retrieved articles for applicability using a coding sheet adapted from Law et al., *2011 *[10]. The selected coding framework utilizes a thoroughly developed taxonomy for health system topics [[10], Lavis et al. unpublished manuscript]. It is used to code health systems evidence on McMaster Health Forum, which is a continuously updated repository of syntheses of research evidence about governance, financial and delivery arrangements within health systems, and about implementation strategies that can support change in health systems [32]. Sub-categories of this coding framework were iteratively developed and refined based on broad categorization schemes such as WHO's "building blocks of health systems" [1] and based on experts' knowledge of health systems and practical experiences with applying the taxonomy [Lavis J et al. Enhancing the Retrieval of Systematic Reviews that Can Inform Health System Management and Policymaking. Unpublished manuscript]. This coding framework has been previously implemented for coding systematic reviews that address health system topics in countries from the region and in low- middle income countries as well [[10,11] Lavis J et al. Enhancing the Retrieval of Systematic Reviews that Can Inform Health System Management and Policymaking. Unpublished manuscript].

The coding sheet was adapted to fit the context of the region. It included themes pertaining to Governance Arrangements, Financial Arrangements, Delivery Arrangements, and Implementation Strategies. Governance Arrangements included codes relating to Policy authority, Organizational authority, Commercial authority, Professional authority, and Consumer & stakeholder involvement. Financial arrangements covered areas including: Financing, Funding, Remuneration, Financial incentives for patients, and Resource allocation. Delivery Arrangements covered several topics mainly: To whom care is provided & with what efforts to reach them, By whom care is provided, Where care is provided, With what information & communication technology (ICT) is care provided, and With what level of quality & safety is care provided. Implementation Strategies comprised two main themes: Consumer-targeted strategies and Provider-targeted strategies. This coding sheet had a total of 116 codes within the above four themes. The complete coding sheet with sub-themes (and results by country) is included in Additional File 1.

Coding was done by two independent reviewers and then checked independently by a third reviewer. The third reviewer checked agreement between the two reviewers and resolved any disagreements. Results of the third review, which encompasses results from the first and second review, are detailed in this article. All reviewers were trained to conduct the review process and were well-informed about HPSR domains included in the coding sheet. The coding sheet was constructed on MS Excel. All reviewers used this standard sheet for data entry. MS Excel was also used for compiling data from each of the 12 countries and for the analysis component and table construction.

To assess the extent to which the existing HPSR that is produced in the region addresses regional priorities pertaining to Health Financing, Human Resources for Health and the Role of the Non-State Sector, a fourth independent reviewer (different from the first three) assumed the task of matching each of the identified articles from the 12 countries with one or more regional priorities. Each article was separately assessed to determine whether the evidence provided in the article can provide or yield information that can address one or more of the regional priorities. The matching exercise was also done using MS Excel.

## Results

As indicated in Table 1, the total articles that fit criteria comprise 11.96% of the 12,488 articles identified in the search. Articles that fit criteria are those that were coded under one or more items in the coding sheet. The countries with the largest number of articles fitting coding criteria coming from Syria (36.94%), Bahrain (33.04%) and Palestine (24.32%) (Table 1). Countries with the fewest articles fitting coding criteria were Egypt (7.02%), Tunisia (7.40%), and Libya (9.94%). It was interesting to observe that countries with the largest number of articles identified through the search strategy had the fewest number of articles fitting criteria.

**Table 1:** Articles fitting coding criteria

Country	Total hits	Fitting criteria	% Fit
Syria	222	82	36.94%

Bahrain	227	75	33.04%

Palestine	366	86	23.50%

Yemen	388	71	18.30%

Jordan	1,424	209	14.68%

Lebanon	1,120	163	14.55%

Oman	548	72	13.14%

Sudan	1,524	186	12.20%

Morocco	1,407	142	10.09%

Libya	905	90	9.94%

Tunisia	1,920	142	7.40%

Egypt	2,437	171	7.02%

**Total**	**12,488**	**1,489**	**11.92%**

**Figure 1:**
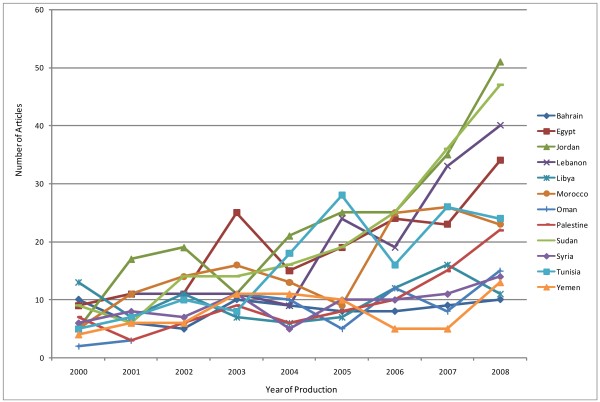
**Annual Production of Articles (fitting coding criteria)**.

Figure 1 details the annual production of articles fitting the criteria in our coding sheet. Results show that countries with a steady increase of production of HSR articles were Jordan, Egypt, Lebanon, Sudan and Tunisia. The increase was highly pronounced after 2005 whereby production of articles spiked for several countries. For other countries, the increase was more pronounced in 2006 while other countries experienced no major change (Figure 1).

Table 2 details the outcome of the coding process across countries for each of the themes listed in the methods section. Articles focusing on Delivery Arrangements comprised 68.1% of total articles fitting criteria followed by 24.4% under Implementation Strategies. Articles under Governance Arrangements comprised only 10% of all articles whereas those classified under Financial Arrangements comprised only 2.1% (Table 2). Additional File 1 lists detailed information on the sub-codes within the below referenced themes and sub-themes.

**Table 2:** Themes by country

	Governance arrangements	Financial arrangements	Delivery arrangements	Implementation strategies	Non-relevant study but with "Health System and Policy' implications
	N (%)	N (%)	N (%)	N (%)	N (%)
Total	149 (10)	31 (21.1)	1014 (68.1)	363 (24.4)	453 (30.4)

Country Specific					
Bahrain	11 (14.7)	0 (0.0)	33 (44)	0 (0.0)	35 (46.7)
Egypt	18 (10.5)	4 (2.3)	81 (47.4)	56 (32.7)	86 (50.3)
Jordan	12 (5.7)	0 (0.0)	140 (67)	64 (30.6)	39 (18.7)
Lebanon	18 (11%)	1 (0.6)	112 (68.7)	37 (22.7)	51 (31.3)
Libya	3 (3.3)	0 (0.0)	89 (98.9)	10 (11.1)	19 (21.1)
Morocco	12 (8.5)	5 (3.5)	114 (80.3)	30 (21.1)	39 (27.5)
Oman	12 (16.7)	5 (6.9)	67 (93.1)	22 (30.6)	10 (13.9)
Palestine	10 (11.6)	4 (4.7)	32 (37.2)	24 (27.9)	36 (41.9)
Sudan	31 (16.7)	5 (2.7)	105 (56.5)	63 (33.9)	49 (26.3)
Syria	4 (4.9)	2 (2.4)	66 (80.5)	19 (23.2)	31 (37.8)
Tunisia	17 (12)	4 (2.8)	101 (71.1)	22 (15.5)	47 (33.1)
Yemen	1 (1.4)	1 (1.4)	74 (104.2)*	16 (22.5)	11 (15.5)

* The same article can be categorized under different subthemes within the same theme, which can result in percentages exceeding 100%.

In terms of Governance Arrangements, most codes focused on Professional authority whereby 15.3% of articles in Oman and 13.4% of those in Sudan were classified under these codes and specifically under the theme on training and licensure. A total of 2.8% of articles in Morocco were coded under Policy authority and specifically stewardship of the non-state sector. In Lebanon, 2.5% of articles fell under the theme of Organizational authority mainly within Accreditation. As for Bahrain, 2.7% of articles were coded under Commercial authority with a focus on licensure/registration and marketing. In Palestine, 2.3% of articles were coded as Consumer and stakeholder involvement specifically Consumer participation in policy and organizational decisions and in service delivery (Table 2, Additional File 1).

Coming to Financial Arrangements, it can be observed that 3.5% of articles in Palestine were coded under Financing with a specific focus on user fees. In Oman, 2.8% of articles were coded within funding with a focus on fee for service and prospective payments. As for Sudan, 2.2% of articles were coded under Resource allocation specifically with lists of suitable and unsuitable products and services (Table 2, Additional File 1).

When it came to Delivery Arrangements, 78.9% of articles in Libya, 55.6% in Oman and 51.2% in Syria were coded under "To whom care is provided." The majority of articles in all three countries were coded under health status and wellness. Articles focusing on providers (by whom care is provided) comprised 17.3% in Yemen (performance management and support), 14.4% in Jordan (focus on provider satisfaction, health and safety, and support), and 12.9% in Lebanon (focus on recruitment and retention, and need, demand and supply). Site of care (where care is provided) was the focus of 13.3% of articles in Bahrain, 12% in Tunisia and 11% in Syria. The focus of articles in all three countries was on primary healthcare. Articles focusing on information technology (with what information and communication technology care is provided) were found to be highest in Oman (12.5%), Morocco (12%), Libya (11.1%) and Jordan (11%). Articles in Jordan, Oman and Morocco focused on Innovation and Research whereas those in Libya focused on Diagnostic medical equipment. As for the theme of quality and safety, this was the focus of 14.1% of articles in each of Morocco and Syria, 11.1% of articles in Oman, and 10.7% of articles in Yemen (Table 2, Additional File 1). Articles in Morocco focused on Quality monitoring systems, those in Oman focused on safety monitoring systems and quality, whereas those in Syria and Yemen focused on regulatory interventions.

In terms of Implementation Strategies, Consumer- targeted strategies were the focus of 26.3% of articles in Egypt and 19.4% in Oman with a focus on information and education provision. Provider- targeted strategies were found to be the focus of 17.2% of articles in Sudan, 14% of those in Palestine and 13.4% of those in Jordan (Table 2, Additional File 1) specifically educational materials for providers.

It is interesting to observe that the proportion of articles classified by reviewers as non-relevant but with health policy and system implications amounted to 50.3% in Egypt, 46.7% in Bahrain and 37.8% in Syria. These articles could not be coded under a specific theme given that they are more public-health related with a focus on epidemiology or country wide surveys but with implications on health systems (Table 2, Additional File 1).

Table 3 details how many of the identified HPSR articles fitting coding criteria provide or yield information that can address regional priorities pertaining to three themes as identified in an earlier study. Within the theme on Health Financing, most articles provided information on population health status and needs (11.1% in Libya and 11% in Jordan). In Yemen, only 4 articles (5.6%) focused on household ability to pay for healthcare whereas only 3 articles (4.2%) shed some light on elements of an equitable health financing system. In Oman, 2 articles (2.8%) provided information on linkages between population health needs and health spending. The Role of Social Health Insurance in guaranteeing equity was highlighted by only 4 articles (2.8%) in each of Morocco and Tunisia. Finally, only 4 articles (2.8%) in Morocco provided information on best practices to develop a national social health insurance system (Table 3).

**Table 3:** Gap analysis of HPSR production matched with regional research priorites identified in El-Jardali et al. 2010 [8]

		Country Specific											
		
Regional Priorities	Total (n = 1,489)	Bahrain (n = 75)	Egypt (n = 171)	Jordan (n = 209)	Lebanon (n = 163)	Libya (n = 90)	Morocco (n = 142)	Oman (n = 72)	Palestine (n = 86)	Sudan (n = 186)	Syria (n = 82)	Tunisia (n = 142)	Yemen (n = 71)
**Health Financing**	**%**	**%**	**%**	**%**	**%**	**%**	**%**	**%**	**%**	**%**	**%**	**%**	**%**

Household ability to pay for health care	1.9%	0.0%	1.8%	3.8%	1.8%	0.0%	0.7%	0.0%	3.5%	2.2%	1.2%	1.4%	5.6%

Elements of an equitable health financing system	1.7%	0.0%	2.3%	1.4%	1.8%	0.0%	1.4$	0.0%	2.3%	1.6%	1.2%	2.8%	4.2%

Role of the Social Health Insurance system in guaranteeing equity	1.0%	0.0%	1.8%	0.5%	0.6%	1.1%	2.8%	0.0%	1.2%	0.0%	0.0%	2.8%	0.0%

Identifying best practices to develop and implement a national social health insurance system	0.5%	0.0%	0.6%	0.0%	0.0%	0.0%	2.8%	1.4%	1.2%	0.0%	0.0%	0.7%	0.0%

Linking population health needs to health spending	0.5%	0.0%	0.0%	0.5%	0.0%	1.1%	0.0%	2.8%	2.3%	0.0%	0.0%	0.7%	0.0%

Clarifying functions and coordination processes between ministries (for example the Ministries of Health and Finance) to improve health system financing and quality of services	0.1%	1.3%	0.0%	0.0%	0.0%	0.0%	0.0%	1.4%	0.0%	0.0%	0.0%	0.0%	0.0%

Means to track financial resources invested in health care to ensure value for money	0.1%	0.0%	0.0%	0.0%	0.0%	0.0%	0.0%	1.4%	0.0%	0.0%	0.0%	0.0%	0.0%

Accurate estimation of the health expenditure from the public and the private sectors including out-of-pocket expenditure	0.0%	0.0%	0.0%	0.0%	0.0%	0.0%	0.0%	0.0%	0.0%	0.0%	0.0%	0.0%	0.0%

Population health status and needs	6.1%	8.0%	2.3%	11.0%	3.1%	11.1%	3.5%	0.0%	9.3%	5.9%	4.9%	5.6%	9.9%

**Total**	**12.0%**	**9.3%**	**8.8%**	**17.2%**	**7.4%**	**13.3%**	**11.3%**	**6.9%**	**19.8%**	**9.7%**	**7.3%**	**14.1%**	**19.7&**

**Human Resources for Health**	**%**	**%**	**%**	**%**	**%**	**%**	**%**	**%**	**%**	**%**	**%**	**%**	**%**

Gaps in existing education and training programs	11.1%	22.7%	14.6%	17.7%	3.7%	6.7%	5.6%	8.3%	12.8%	6.5%	9.8%	10.6%	19.7%

Ways that can enable education and training programs to meet the population health needs	9.0%	4.0%	13.5%	9.1%	10.4%	1.1%	10.6%	6.9%	8.1%	12.4%	3.7%	9.2%	7.0%

Methods to measure HRH performance and productivity	8.3%	30.7%	9.4%	9.6%	7.4%	1.1%	6.3%	11.1%	8.1%	3.8%	3.7%	8.5%	7.0%

Ways to improve staff satisfaction	2.6%	2.7%	4.1%	5.7%	3.1%	0.0%	2.1%	2.8%	1.2%	0.0%	0.0%	3.5%	1.4%

Elements of performance evaluation	2.4%	5.3%	1.8%	2.4%	3.1%	0.0%	1.4%	5.6%	0.0%	0.5%	0.0%	7.7%	1.4%

Develop incentive mechanisms to better-manage the existing stock of HRH	2.1%	0.0%	2.9%	7.7%	4.3%	1.1%	0.7%	0.0%	0.0%	0.0%	0.0%	1.4%	0.0%

Information on patient satisfaction	1.9%	0.0%	4.1%	1.9%	0.6%	0.0%	3.5%	1.4%	3.5%	1.1%	0.0%	2.8%	2.8%

Accurate estimates and needs in numbers and specialties (mapping)	0.9%	1.3%	0.0%	1.0%	1.2%	0.0%	1.4%	1.4%	3.5%	0.0%	0.0%	1.4%	0.0%

Develop simulation models for HRH planning	0.5%	0.0%	0.0%	0.5%	0.6%	0.0%	0.0%	1.4%	3.5%	0.0%	0.0%	0.7%	0.0%

Means to develop HRH information systems in ministries of health and national observatories	0.2%	1.3%	0.0%	0.0%	0.6%	0.0%	0.0%	1.4%	0.0%	0.0%	0.0%	0.0%	0.0%

**Total**	**39.0%**	**68.0%**	**50.3%**	**55.6%**	**35.0%**	**10.0%**	**31.7%**	**40.3%**	**40.7%**	**24.2%**	**17.1%**	**45.8%**	**39.4%**

**Role of the Non-State Sector**	**%**	**%**	**%**	**%**	**%**	**%**	**%**	**%**	**%**	**%**	**%**	**%**	**%**

Ways to regulate and monitor the quality of care in the private sector	0.9%	1.3%	1.8%	1.0%	2.5%	0.0%	0.7%	0.0%	0.0%	0.0%	1.2%	0.7%	0.0%

Ways to optimize the use of the existing resources of the non-state sector to meet health system objectives	0.8%	1.3%	1.2%	0.5%	1.2%	0.0%	1.4%	0.0%	0.0%	0.0%	1.2%	0.7%	2.8%

Ways for the public and private sector to complement their service delivery	0.8%	1.3%	0.6%	0.5%	1.8%	0.0%	0.7%	1.4%	0.0%	0.5%	1.2%	1.4%	0.0%

Scope, resources and kind of services provided by the non-state sector	0.8%	1.3%	0.6%	0.5%	0.6%	0.0%	1.4%	0.0%	1.2%	0.5%	1.2%	0.7%	2.8%

Defining the role and responsibility of the non-state sector	0.7%	1.3%	0.6%	0.5%	1.2%	0.0%	0.7%	0.0%	1.2%	0.5%	1.2%	0.7%	0.0%

Areas where the state and civil society groups can complement each other	0.6%	0.0%	0.6%	0.0%	0.6%	0.0%	1.4%	0.0%	3.5%	0.0%	0.0%	0.0%	2.8%

Foundation/elements for building strong public-private partnerships	0.6%	1.3%	0.6%	0.5%	1.2%	0.0%	0.7%	0.0%	0.0%	0.5%	1.2%	0.7%	0.0%

Ways to develop effective contracting mechanisms with the private and other non-state sectors	0.6%	1.3%	0.6%	0.5%	1.2%	0.0%	0.7%	0.0%	0.0%	0.5%	1.2%	0.7%	0.0%

Accreditation standards for private sector	0.2%	0.0%	0.0%	0.0%	1.8%	0.0%	0.0%	0.0%	0.0%	0.0%	0.0%	0.0%	0.0%

Measuring client satisfaction	0.1%	0.0%	0.6%	0.5%	0.0%	0.0%	0.0%	0.0%	0.0%	0.0%	0.0%	0.0%	0.0%

National plan for the contribution of the non-state sector	0.0%	0.0%	0.0%	0.0%	0.0%	0.0%	0.0%	0.0%	0.0%	0.0%	0.0%	0.0%	0.0%

National database on the non-state sector	0.0%	0.0%	0.0%	0.0%	0.0%	0.0%	0.0%	0.0%	0.0%	0.0%	0.0%	0.0%	0.0%

**Total**	**6.1%**	**9.3%**	**7.0%**	**4.3%**	**12.3%**	**0.0%**	**7.7%**	**1.4%**	**5.8%**	**2.7%**	**8.5%**	**5.6%**	**8.5%**

Do not fit under any of the priorities	67.8%	44.0%	66.1%	55.5%	71.2%	82.2%	71.1%	76.4%	67.4%	76.3%	81.7%	65.5%	59.2%

Results show that many of the coded articles can provide or yield information to address some priorities pertaining to Human Resources for Health (HRH). This is not surprising since the majority of articles were coded under Delivery Arrangements. Measuring HRH productivity was the focus of 30.7% of articles in Bahrain. A total of 22.7% of articles in Bahrain, 19.7% in Yemen and 17.7% in Jordan focused on gaps in existing education and training programs. Enabling education and training programs to meet population health needs was the focus of 13.5% of articles in Egypt and 12.4% of articles in Sudan. Elements of performance evaluation were highlighted in only 4 articles (5.6%) in Oman and 4 articles (5.3%) in Bahrain. A total of 7.7% of articles in Jordan focused on developing incentives to better manage the existing stock of HRH. Improving staff satisfaction was the focus of 5.7% of articles in Jordan and 4.1% of articles in Egypt. It is worth noting that articles highlighting the means to develop an HRH information system and national observatories was only highlighted in one article in Bahrain (1.3%) and 1 article in Oman (1.4%) (Table 2).

Few articles focused on the Role of the Non-State Sector. Only 3 articles (3.5%) in Palestine focused on where the state and civil society can complement each other. Ways to regulate and monitor quality of care in the private sector was the focus of 4 articles (2.5%) in Lebanon. Optimizing the use of existing resources of the non-state sector to meet health system objectives was the focus of 2 articles (2.8%) in Yemen. Finding ways for the public and private sector to complement each other was the focus of 3 articles (1.8%) in Lebanon, and 2 articles (1.4%) in each of Oman and Tunisia (Table 3).

A total of 81.7% of articles in Syria did not meet any of the listed priorities; neither did 76.4% of articles in Oman and 76.3% of those in Sudan. It is worth noting here that the articles do not necessarily provide exact answers to meet the highlighted priorities but they might to a certain extent, yield information that would help address them.

## Discussion

### Principal Findings

There has been an increase in the production of HPSR over the years, especially for Jordan, Egypt, Lebanon, Sudan and Tunisia. The increase in HPSR production peaked after the year 2005 for most countries. This is an important finding as it suggests that the surge in HPSR production in the EMR was probably influenced by international calls to support HPSR and its use in policymaking that were issued during that time at the Mexico Summit in 2004 [3] and the World Health Assembly Resolution in 2005 [4]. Notably, countries with the largest number of identified articles had the fewest number of articles fitting criteria for HPSR. This shows that although countries from the region may be producing a significant number of research articles; however, these may not necessarily address health policy and systems. Overall, Jordan accounted for more HPSR articles than any other country from the region followed by Sudan and Egypt. While Oman, Bahrain, Yemen and Syria accounted for the least HPSR production.

Most of the identified articles focused on Delivery arrangements (68.1%), followed by Implementation Strategies (24.4%), Governance Arrangements (10.0%) and Financial Arrangements (2.1%). This clearly indicates the gaps in the production of HPSR evidence in the EMR. Among the articles that focused on delivery arrangements, many of them (36.5%) covered the category "to whom care is provided and with what efforts to reach them".

In mapping out which aspects of the previously indentified priorities are already addressed by existing research, most of the HPSR articles that we found in selected EMR countries address priorities in Human Resources for Health, with most articles providing information on gaps in existing education and training programs. This is expected since most of the articles covered the theme delivery arrangement. Several articles match in a general way some of the regional priorities within the three themes; however, this does not necessarily mean that these articles provide specific evidence for informing decisions on these priorities, but rather they may yield information that would help address those priorities.

### Strengths and limitations

To our knowledge, this is the first stocktaking paper of HPSR production and gaps in the EMR. Due to limitations with time and resources, this assessment only includes articles from the year 2000 to 2008. Still, it is a first attempt to map out HPSR production in the region and conduct a gap assessment. In this study, specific inclusion criteria was used to select HPSR in the region from the Medline database. This analysis will be repeated in the coming years to show trends and explore alignments between demand for evidence from policy makers and supply of HPSR research from researchers. In future studies, we will also aim to include all EMR countries.

Our study has few limitations. We used the Medline database for searching for articles from the 12 study countries, and the publications indexed in Medline might not fully reflect the extent of EMR output from the selected countries. It should be acknowledged that some researchers in the region may be publishing their research papers in local or regional journals in the Middle East which are not indexed in Medline or might not even be available online. While Medline can capture some articles published in Arabic or French, many other articles published in local languages and often highlight country specific studies cannot be captured given that the journals are not indexed on Medline. In most cases, many of the articles indexed in Arabic or French for the 12 countries rarely had abstracts or links to full text articles. We attempted to secure links to full text through other online sources but could not in most cases. As such, most of these articles were not included but we did take note of the authors and sources of these papers to try to secure links to full text in the future. In short, despite the first that knowledge in the form of unpublished studies, commissioned reports, etc. is not captured in our stocktaking exercise, this study is a first attempt to map out HPSR production in the region and conduct a gap assessment. Moreover, a separate strategy accounting for the complexities of search options for grey literature (available online and through non-internet sources), international organizations, researchers, funding bodies and the issue of non-publicly accessible reports will be developed. Given that such information is not readily available and takes a longer time, this component is still in progress.

While Medline is a major source of articles that are published on HPSR in the region, it is not the only one. EMBASE also indexes journals that might include articles from the 12 countries and beyond. It should also be noted though that EMBASE indexes articles that might also have been indexed on Medline and although an option exists to exclude these articles from an advanced search, some articles already indexed on Medline might be retrieved. In fact, the overlap between Medline and EMBASE can range between 10 and 87% depending on the topic [33]. Evidence comparing the two databases also indicates that Medline outperforms EMBASE [33]. Assessment of search strategies for HPSR using Medline showed that the database offers high sensitivity and specificity but has low precision given the low production of such articles [34]. In future studies, we plan to conduct a similar search for the 12 study countries on EMBASE making sure to account for the duplication between the two databases so we can complement the findings from Medline and provide a more comprehensive outlook on HPSR production in the region. We also plan to expand the search to include countries excluded from this assessment, particularly Pakistan and Iran which produce a lot of research pertaining to HPSR.

Although study findings may not reflect the context of all EMR countries, the results of this research are a first essential step towards assessing existing research production and identifying gaps in 12 of the 22 countries in the region. Lessons learned from this exercise could be extrapolated in future studies assessing HPSR production in the region. Moreover, and given that many of the study countries are members of EVIPNet EMR, this can provide an opportunity for the KT platforms to adopt this agenda and to remedy the mis-match between research production and priorities taking their own contextual factors into account.

It is also worth highlighting that the priority setting exercise which was used in Table 3 included 9 countries. Only 8 of these countries were included in this assessment. However, this does not necessarily indicate that these priorities are not applicable to all countries. In fact, countries that were not included in the priority setting exercise (Bahrain, Libya, Oman and Sudan) did produce articles on several of these priorities. This might indicate that these are priority issues of importance in their context as well. Another limitation pertains to the fact that the priorities identified in the previous priority setting exercise do not encompass all health systems priorities in the region, since the original exercise was focused only on three thematic priority areas. Lastly, the stocktaking exercise covers HPSR production from Medline only for years 2000-2008. We assume additional HPSR was produced in 2009-2011 and this will be captured in future studies. At the same time, we believe that the 8 years review we conducted provides a good overview of the nature of production and gaps.

### Findings in Relation to Other Studies

Gaps in the production of HPSR from the region are paralleled by low production of systematic reviews that address health systems topics. A recent exercise that profiled the production of systematic reviews in the region revealed that only seven of 384 identified reviews addressed health systems topics [10,11]. Furthermore, a print media analyses exercise showed that the region is among the lowest in terms of the articles that describe or use health research evidence [9].

Our findings regarding the lack of HPSR to address priority issues, especially in health financing and the role of the non-state sector further corroborate those reported by health policymakers in the EMR in a recent survey about the use of research evidence, whereby the majority reported lack of policy relevant research to inform decision-making [El-Jardali et al. Use of Health Systems Evidence by Policymakers in Eastern Mediterranean Countries: Views, Practices, and Contextual Influences under review].

The limited production of research in the EMR is not only related to HPSR. Studies that profiled on the production of biomedical research in countries from the region also reported lag in research production, with Egypt and Jordan also leading the production of biomedical research in the Arab countries from the EMR [17,19-23].

The literature cites several factors leading to the lag in research production in the EMR. For instance, health policy and systems researchers from the region reported lack of funding and investment in priority health research in addition to the lack of policymakers' involvement in setting clear priorities for health systems and policy research [El-Jardali et al. Increasing the Use of Health Systems and Policy Research Evidence in the Health Policymaking in Eastern Mediterranean Countries: Views and Practices of Researchers, under review]. Furthermore, the literature on research analysis (not necessarily HPSR) reports several challenges to research production in countries from the region. Regional conflicts and political instability, lack of funding, as well as the difficulty of publishing research of local interest in high impact journals were reported as major barriers for the production of research in the region [13,19,20,35,36]. Weakness of democratic institutions was also reported to restrict free scientific inquiry and access to information and contributed to the migration of researchers from the region [36]. Furthermore, some higher academic institutions lack basic requirements for supporting researchers in conducting research, such as institutional review boards and grants and financial units that can deal with managing grant funds [36]. Furthermore, the criteria for promotion of researchers vary across institutions, for some universities academic research production is not considered a priority for career advancement, and this might de-motivate researchers from producing high quality research [37].

### Implications for Policy and Research

This study provides baseline assessment of HPSR production in the region, and the methodology can be used in future studies to analyze trends in the production of HPSR over time. Our study provides an HPSR research agenda which would help assist researchers in identifying areas for research.

Health policy and systems researchers in the EMR should also be trained on increasing the relevance of their research including focusing on priority policy issues. Efforts should be directed towards building the capacity of researchers in producing HPSR including systematic reviews to help policymakers make evidence-informed decisions as well as in raising the awareness of policymakers to seek, commission, and fund research to inform policy priorities [El-Jardali et al. Use of Health Systems Evidence by Policymakers in Eastern Mediterranean Countries: Views, Practices, and Contextual Influences under review; El-Jardali et al. Increasing the Use of Health Systems and Policy Research Evidence in the Health Policymaking in Eastern Mediterranean Countries: Views and Practices of Researchers, under review]. Efforts should also be directed towards accessing tacit knowledge to guide policymaking in addition to tapping on HPSR that exist in the grey literature in the EMR. Academic and research institutes, as well as WHO EMRO, research funding agencies, and national policymakers can all support the production of HPSR by increasing funding and investments in health research, setting clear priorities for research, and by providing incentive for researchers to produce quality HPSR for informing health policymaking [[14], El-Jardali et al. Increasing the Use of Health Systems and Policy Research Evidence in the Health Policymaking in Eastern Mediterranean Countries: Views and Practices of Researchers, under review]. Our findings suggest that there is a need for increased funding for HPSR in the region. Funding bodies can also play a major role in enhancing the production of HPSR in the EMR. It is hoped that funding agencies and countries will support and align financial and human resources towards addressing the gaps in HPSR that have been identified. And funding for HPSR should become aligned with national and regional priorities. Innvær *et al*. (2002) [38] reported that the direct use of research evidence is greatest in the case of commissioned research to fill a knowledge gap identified by stakeholders.

Existing research that addresses regional priorities is limited. Our findings suggest that primary HPSR is required. Due to time constraints, it is essential that some of those regional priorities are swiftly answered through research synthesis. Given the HPSR in the region, there is a need to look at how to assess the relevance and applicability of the international body of research evidence to the policy concerns and priorities identified in the region. Using and customizing findings systematic reviews may play a role in informing policy and decision-making in health systems of the EMR.

## Conclusion

Knowledge translation requires the availability of context specific knowledge that addresses policy priority areas. Despite global calls for producing and translating HPSR into policy, there is still significant gap in the production of HPSR in the EMR. More efforts are needed to produce HPSR and to align production and translation with the demand for evidence from policymakers.

Findings provide baseline assessment against which to compare the future production of HPSR in the region and at the country level and guide future work on strengthening evidence- informed policies in the region. Study findings can also help inform and direct future plans and activities for the EVIPNet EMR, WHO EMRO, and Middle East and North Africa (MENA) Health Policy Forum, in addition to being useful for countries that host or are planning to host KT platforms in the region.

## List of Abbreviations

EMR: Eastern Mediterranean Region; EVIPNet EMR: Evidence Informed Policy Network- Eastern Mediterranean Region; HPSR: Health Policy and Systems Research; HRH: Human Resources for Health; ICT: Information & Communication Technology; LMIC: Low and Middle Income Countries; MENA: Middle East and North Africa; MeSH: Medical Subject Headings; MOH: Ministries of Health; WHO EMRO: Eastern Mediterranean Regional Office of the World Health Organization; WHO: World Health Organization.

## Competing interests

The authors declare that they have no competing interests.

## Authors' contributions

FE made substantial contributions to the conception, design, analysis and interpretation of results and write-up of the manuscript. DJ made substantial contributions to design, acquisition, review of articles, analysis of data, interpretation of results and write-up of findings. NA contributed to review of articles, data analysis and tabulation in addition to write up of the paper. MJ made substantial contribution review of articles, analysis of data and revising the intellectual content of the paper. SR, CM, SM and CS contributed revision of articles and write-up of the final version of the paper. All authors read and approved the final manuscript.

## Supplementary Material

Additional file 1 **Detailed Coding Sheet with Country Specific Results**. contains country specific results within each theme and topic. Click here for file
