# Supplementary material for: Health Policy and Systems Research in Twelve Eastern Mediterranean Countries: a stocktaking of production and gaps (2000-2008)
Source: Health Res Policy Syst. 2011 Oct 7;9:39. doi: 10.1186/1478-4505-9-39 (PMC3224558; doi:10.1186/1478-4505-9-39)
Supplement: Additional file 1 — Detailed Coding Sheet with Country Specific Results. contains country specific results within each theme and topic. [file 1478-4505-9-39-S1.PDF]

Additional File 1: Detailed Coding sheet with country specific results

			Bahrain		Egypt		Jordan		Lebanon		Libya		Morocco		Oman		Palestine		Sudan		Syria		Tunisia		Yemen	
			N	%	N	%	N	%	N	%	N	%	N	%	N	%	N	%	N	%	N	%	N	%	N	%
Governance arrangements	Policy Authority	Centralization / decentralization of policy authority	0	0.0	0	0.0	0	0.0	0	0.0	0	0.0	0	0.0	0	0.0	0	0.0	1	20.0	0	0.0	0	0.0	0	0.0%
		Stewardship of the non-state sector's role in financing & delivering	1	100	1	50.0	1	100	3	75.0	0	0.0	3	75.0	0	0.0	1	50.0	1	20.0	1	50.0	1	33.3	0	0.0%
		Coverage/reimbursement decision-making authority about what or who is covered	0	0.0	1	50.0	0	0.0	1	25.0	0	0.0	1	25.0	0	0.0	1	50.0	3	60.0	1	50.0	2	66.7	0	0.0%
		Corruption protections	0	0.0	0	0.0	0	0.0	0	0.0	0	0.0	0	0.0	0	0.0	0	0.0	0	0.0	0	0.0	0	0.0	0	0.0%
		**Total Policy Authority**	1	1.3	2	1.2	1	0.5	4	2.5	0	0.0	4	2.8	0	0.0	2	2.3	5	2.7	2	2.4	3	2.1	0	0.0%
	Organizational authority	Ownership	0	0.0	0	0.0	0	0.0	0	0.0	0	0.0	0	0.0	0	0.0	0	0.0	0	0.0	0	0.0	0	0.0	0	0.0%
		Accreditation	0	0.0	0	0.0	0	0.0	3	75.0	0	0.0	0	0.0	0	0.0	0	0.0	0	0.0	0	0.0	0	0.0	0	0.0%
		Networks / multi-institutional arrangements	0	0.0	0	0.0	0	0.0	0	0.0	0	0.0	0	0.0	0	0.0	0	0.0	0	0.0	0	0.0	0	0.0	0	0.0%
		Strategic management	0	0.0	1	100	0	0.0	1	25.0	0	0.0	1	100	0	0.0	1	100	0	0.0	0	0.0	0	0.0	0	0.0%
		**Total Organizational Authority**	0	0.0	1	0.6	0	0.0	4	2.5	0	0.0	1	0.7	0	0.0	2	2.3	0	0.0	0	0.0	0	0.0	0	0.0%
	Commercial authority	Licensure / registration	1	50.0	0	0.0	0	0.0	0	0.0	0	0.0	0	0.0	0	0.0	0	0.0	0	0.0	0	0.0	0	0.0	0	0.0%
		Patents & profits	0	0.0	0	0.0	0	0.0	0	0.0	0	0.0	0	0.0	0	0.0	0	0.0	0	0.0	0	0.0	0	0.0	0	0.0%
		Pricing & purchasing	1	50.0	0	0.0	0	0.0	0	0.0	0	0.0	0	0.0	1	100	1	100	0	0.0	0	0.0	0	0.0	0	0.0%
		Marketing	0	0.0	0	0.0	0	0.0	0	0.0	0	0.0	0	0.0	0	0.0	0	0.0	0	0.0	0	0.0	0	0.0	0	0.0%
		Sales & dispensing	0	0.0	0	0.0	0	0.0	0	0.0	0	0.0	0	0.0	0	0.0	0	0.0	1	100	0	0.0	0	0.0	0	0.0%
		Commercial liability	0	0.0	0	0.0	0	0.0	0	0.0	0	0.0	0	0.0	0	0.0	0	0.0	0	0.0	0	0.0	0	0.0	0	0.0%
		**Total Commercial Authority**	2	2.7	0	0.0	0	0.0	0	0.0	0	0.0	0	0.0	1	1.4	1	1.2	1	0.5	0	0.0	0	0.0	0	0.0%
	Professional authority	Training / licensure / registration	5	33.3	12	80.0	5	45.5	6	60.0	1	33.3	6	85.7	5	45.5	2	66.7	12	48.0	1	50.0	12	85.7	1	100%
		Scope of practice	0	0.0	0	0.0	1	9.1	0	0.0	0	0.0	0	0.0	0	0.0	0	0.0	0	0.0	0	0.0	1	7.1	0	0.0%
		Location of	0	0.0	0	0.0	0	0.0	1	10.0	0	0.0	0	0.0	0	0.0	0	0.0	0	0.0	0	0.0	0	0.0	0	0.0%

			Bahrain		Egypt		Jordan		Lebanon		Libya		Morocco		Oman		Palestine		Sudan		Syria		Tunisia		Yemen	
			N	%	N	%	N	%	N	%	N	%	N	%	N	%	N	%	N	%	N	%	N	%	N	%
		practice																								
		Single or dual-system practice	0	0.0	0	0.0	0	0.0	0	0.0	0	0.0	0	0.0	0	0.0	0	0.0	0	0.0	0	0.0	0	0.0	0	0.0%
		Content of practice	0	0.0	2	13.3	0	0.0	0	0.0	0	0.0	0	0.0	0	0.0	0	0.0	3	12.0	0	0.0	0	0.0	0	0.0%
		Quality of practice / clinical governance	1	6.7	0	0.0	2	18.2	3	30.0	2	66.7	0	0.0	2	18.2	0	0.0	1	4.0	1	50.0	0	0.0	0	0.0%
		Professional competence	8	53.3	1	6.7	3	27.3	0	0.0	0	0.0	1	14.3	4	36.4	1	33.3	9	36.0	0	0.0	1	7.1	0	0.0%
		Professional liability	1	6.7	0	0.0	0	0.0	0	0.0	0	0.0	0	0.0	0	0.0	0	0.0	0	0.0	0	0.0	0	0.0	0	0.0%
		**Total Professional Authority**	8	10.7	15	8.8	11	5.3	10	6.1	3	3.3	7	4.9	11	15.3	3	3.5	25	13.4	2	2.4	14	9.9	1	1.4%
	Consumer & stakeholder involvement	Consumer participation in policy & organizational decisions	0	0.0	0	0.0	0	0.0	0	0.0	0	0.0	0	0.0	0	0.0	1	50.0	0	0.0	0	0.0	0	0.0	0	0.0%
		Consumer participation in service delivery	0	0.0	0	0.0	0	0.0	0	0.0	0	0.0	0	0.0	0	0.0	1	50.0	0	0.0	0	0.0	0	0.0	0	0.0%
		Consumer complaints management	0	0.0	0	0.0	0	0.0	0	0.0	0	0.0	0	0.0	0	0.0	0	0.0	0	0.0	0	0.0	0	0.0	0	0.0%
		Stakeholder participation in policy & organizational decisions	0	0.0	0	0.0	0	0.0	0	0.0	0	0.0	0	0.0	0	0.0	0	0.0	0	0.0	0	0.0	0	0.0	0	0.0%
		**Total Consumer and Stakeholder Involvement**	0	0.0	0	0.0	0	0.0	0	0.0	0	0.0	0	0.0	0	0.0	2	2.3	0	0.0	0	0.0	0	0.0	0	0.0%
		**Total Governance Arrangements**	11	14.7	18	10.5	12	5.7	18	11.0	3	3.3	12	8.5	12	16.7	10	11.6	31	16.7	4	4.9	17	12.0	1	1.4%
**Financial arrangements**	Financing	Taxation	0	0.0	0	0.0	0	0.0	0	0.0	0	0.0	0	0.0	0	0.0	0	0.0	0	0.0	0	0.0	0	0.0	0	0.0%
		Social insurance	0	0.0	1	50.0	0	0.0	0	0.0	0	0.0	4	100	1	50.0	1	33.3	0	0.0	0	0.0	3	75.0	0	0.0%
		Community-based insurance	0	0.0	1	50.0	0	0.0	0	0.0	0	0.0	0	0.0	0	0.0	0	0.0	0	0.0	0	0.0	1	25.0	0	0.0%
		Private insurance	0	0.0	0	0.0	0	0.0	1	100	0	0.0	0	0.0	0	0.0	0	0.0	0	0.0	0	0.0	0	0.0	0	0.0%
		User fees	0	0.0	0	0.0	0	0.0	0	0.0	0	0.0	0	0.0	1	50.0	2	66.7	1	100	0	0.0	0	0.0	0	0.0%
		Conditional cash transfers	0	0.0	0	0.0	0	0.0	0	0.0	0	0.0	0	0.0	0	0.0	0	0.0	0	0.0	0	0.0	0	0.0	0	0.0%
		**Total Financing**	0	0.0	2	1.2	0	0.0	1	0.6	0	0.0	4	2.8	2	2.8	3	3.5	1	0.5	0	0.0	4	2.8	0	0.0%
	Funding	Fee-for-service	0	0.0	0	0.0	0	0.0	0	0.0	0	0.0	0	0.0	1	50.0	0	0.0	0	0.0	0	0.0	0	0.0	0	0.0%

			Bahrain		Egypt		Jordan		Lebanon		Libya		Morocco		Oman		Palestine		Sudan		Syria		Tunisia		Yemen	
			**N**	**%**	**N**	**%**	**N**	**%**	**N**	**%**	**N**	**%**	**N**	**%**	**N**	**%**	**N**	**%**	**N**	**%**	**N**	**%**	**N**	**%**	**N**	**%**
		Capitation	0	0.0	0	0.0	0	0.0	0	0.0	0	0.0	0	0.0	0	0.0	0	0.0	0	0.0	0	0.0	0	0.0	0	0.0%
		Global budget	0	0.0	0	0.0	0	0.0	0	0.0	0	0.0	0	0.0	0	0.0	0	0.0	0	0.0	0	0.0	0	0.0	0	0.0%
		Prospective payment (for a particular diagnosis, product, etc.)	0	0.0	0	0.0	0	0.0	0	0.0	0	0.0	0	0.0	1	50.0	0	0.0	0	0.0	0	0.0	0	0.0	0	0.0%
		Indicative budgets	0	0.0	0	0.0	0	0.0	0	0.0	0	0.0	0	0.0	0	0.0	0	0.0	0	0.0	0	0.0	0	0.0	0	0.0%
		Targeted payments / penalties	0	0.0	0	0.0	0	0.0	0	0.0	0	0.0	0	0.0	0	0.0	0	0.0	0	0.0	0	0.0	0	0.0	0	0.0%
		**Total Funding**	0	0.0	0	0.0	0	0.0	0	0.0	0	0.0	0	0.0	2	2.8	0	0.0	0	0.0	0	0.0	0	0.0	0	0.0%
	Remuneration	Fee-for-service	0	0.0	0	0.0	0	0.0	0	0.0	0	0.0	0	0.0	0	0.0	0	0.0	0	0.0	0	0.0	0	0.0	0	0.0%
		Capitation	0	0.0	0	0.0	0	0.0	0	0.0	0	0.0	0	0.0	0	0.0	0	0.0	0	0.0	0	0.0	0	0.0	0	0.0%
		Salary	0	0.0	0	0.0	0	0.0	0	0.0	0	0.0	0	0.0	0	0.0	0	0.0	0	0.0	0	0.0	0	0.0	0	0.0%
		Prospective payment	0	0.0	0	0.0	0	0.0	0	0.0	0	0.0	0	0.0	0	0.0	0	0.0	0	0.0	0	0.0	0	0.0	0	0.0%
		Indicative budgets	0	0.0	0	0.0	0	0.0	0	0.0	0	0.0	0	0.0	0	0.0	0	0.0	0	0.0	0	0.0	0	0.0	0	0.0%
		Annual caps on provider income	0	0.0	0	0.0	0	0.0	0	0.0	0	0.0	0	0.0	0	0.0	0	0.0	0	0.0	0	0.0	0	0.0	0	0.0%
		Targeted payments / penalties	0	0.0	0	0.0	0	0.0	0	0.0	0	0.0	0	0.0	0	0.0	0	0.0	0	0.0	0	0.0	0	0.0	0	0.0%
		**Total Remuneration**	0	0.0	0	0.0	0	0.0	0	0.0	0	0.0	0	0.0	0	0.0	0	0.0	0	0.0	0	0.0	0	0.0	0	0.0%
	Financial incentives for patients	Premium	0	0.0	0	0.0	0	0.0	0	0.0	0	0.0	0	0.0	0	0.0	0	0.0	0	0.0	0	0.0	0	0.0	0	0.0%
		Cost-sharing (e.g., co-payment, user fee)	0	0.0	0	0.0	0	0.0	0	0.0	0	0.0	1	100	0	0.0	0	0.0	0	0.0	0	0.0	0	0.0	0	0.0%
		Targeted payments (e.g., conditional cash transfer)/ penalties	0	0.0	0	0.0	0	0.0	0	0.0	0	0.0	0	0.0	0	0.0	0	0.0	0	0.0	0	0.0	0	0.0	0	0.0%
		**Total Financial Incentives for patients**	0	0.0	0	0.0	0	0.0	0	0.0	0	0.0	1	0.7	0	0.0	0	0.0	0	0.0	0	0.0	0	0.0	0	0.0%
	Resource allocation	Changes in scope & nature of benefits & services	0	0.0	1	50.0	0	0.0	0	0.0	0	0.0	0	0.0	0	0.0	1	100	0	0.0	1	50.0	0	0.0	0	0.0

			Bahrain		Egypt		Jordan		Lebanon		Libya		Morocco		Oman		Palestine		Sudan		Syria		Tunisia		Yemen	
			N	%	N	%	N	%	N	%	N	%	N	%	N	%	N	%	N	%	N	%	N	%	N	%
		Lists of covered / reimbursed products & services (e.g., "positive" lists such as formularies & "negative" lists such as restrictions)	0	0.0	0	0.0	0	0.0	0	0.0	0	0.0	0	0.0	0	0.0	0	0.0	0	0.0	0	0.0	0	0.0	0	0.0
		Lists of substitutable products & services	0	0.0	1	50.0	0	0.0	0	0.0	0	0.0	0	0.0	0	0.0	0	0.0	4	100	1	50.0	0	0.0	0	0.0
		Restrictions in coverage / reimbursement rates for covered products & services	0	0.0	0	0.0	0	0.0	0	0.0	0	0.0	0	0.0	0	0.0	0	0.0	0	0.0	0	0.0	0	0.0	0	0.0
		Caps on coverage / reimbursement for covered products & services	0	0.0	0	0.0	0	0.0	0	0.0	0	0.0	0	0.0	0	0.0	0	0.0	0	0.0	0	0.0	0	0.0	0	0.0
		Prior approval requirement	0	0.0	0	0.0	0	0.0	0	0.0	0	0.0	0	0.0	0	0.0	0	0.0	0	0.0	0	0.0	0	0.0	1	100
		Catastrophic drug coverage	0	0.0	0	0.0	0	0.0	0	0.0	0	0.0	0	0.0	1	100	0	0.0	0	0.0	0	0.0	0	0.0	0	0.0
		**Total Resource Allocation**	0	0.0	2	1.2	0	0.0	0	0.0	0	0.0	0	0.0	1	1.4	1	1.2	4	2.2	2	2.4	0	0.0	1	1.4
		**Total Financial Arrangements**	0	0.0	4	2.3	0	0.0	1	0.6	0	0.0	5	3.5	5	6.9	4	4.7	5	2.7	2	2.4	4	2.8	1	1.4
**Delivery arrangements**	To whom care is provided & with what efforts to reach them	Timely access	2	28.6	1	7.1	2	2.5	1	1.5	0	0.0	2	4.0	0	0.0	1	7.7	0	0.0	1	2.4	1	2.1	4	9.5
		Culturally appropriate care	0	0.0	2	14.3	3	3.8	2	2.9	0	0.0	2	4.0	3	7.5	1	7.7	2	3.5	6	14.6	7	14.9	8	19.0
		Case management	5	71.4	3	21.4	37	46.8	24	35.3	15	21.1	26	52.0	10	25.0	0	0.0	31	54.4	12	29.3	20	42.6	22	52.4
		Package of care / care pathways / disease management	0	0.0	2	14.3	1	1.3	2	2.9	3	4.2	4	8.0	3	7.5	0	0.0	1	1.8	8	19.5	5	10.6	1	2.4
		Timely access	0	0.0	0	0.0	0	0.0	0	0.0	0	0.0	0	0.0	0	0.0	1	7.7	0	0.0	0	0.0	0	0.0	1	2.4
		Health status & wellness	2	28.6	7	50.0	38	48.1	40	58.8	53	74.6	18	36.0	24	60.0	11	84.6	23	40.4	15	36.6	15	31.9	10	23.8
		**Total to whom care is provided and with what efforts to reach**	9	12.0	15	8.8	81	38.8	69	42.3	71	78.9	52	36.6	40	55.6	14	16.3	57	30.6	42	51.2	48	33.8	46	64.8

			Bahrain		Egypt		Jordan		Lebanon		Libya		Morocco		Oman		Palestine		Sudan		Syria		Tunisia		Yemen	
			N	%	N	%	N	%	N	%	N	%	N	%	N	%	N	%	N	%	N	%	N	%	N	%
		**them**																								
	By whom care is provided	System - Need, demand & supply	2	28.6	2	14.3	4	13.3	4	19.0	0	0.0	3	21.4	2	33.3	3	60.0	1	20.0	1	25.0	3	37.5	1	7.7
		System - Recruitment, retention & transitions	1	14.3	0	0.0	2	6.7	6	28.6	0	0.0	2	14.3	0	0.0	1	20.0	0	0.0	0	0.0	1	12.5	0	0.0
		System - Performance management	2	28.6	2	14.3	4	13.3	2	9.5	0	0.0	2	14.3	1	16.7	0	0.0	1	20.0	1	25.0	2	25.0	5	38.5
		Workplace conditions – Provider satisfaction	0	0.0	4	28.6	7	23.3	2	9.5	0	0.0	0	0.0	1	16.7	0	0.0	0	0.0	0	0.0	0	0.0	0	0.0
		Workplace conditions – Health & safety	1	14.3	4	28.6	5	16.7	3	14.3	3	100	7	50.0	0	0.0	0	0.0	2	40.0	0	0.0	2	25.0	2	15.4
		Skill mix – Role performance	0	0.0	0	0.0	3	10.0	0	0.0	0	0.0	0	0.0	0	0.0	0	0.0	0	0.0	0	0.0	0	0.0	0	0.0
		Skill mix – Role expansion or extension	0	0.0	0	0.0	0	0.0	0	0.0	0	0.0	0	0.0	0	0.0	0	0.0	1	20.0	0	0.0	0	0.0	0	0.0
		Skill mix - Substitution	0	0.0	0	0.0	0	0.0	0	0.0	0	0.0	0	0.0	0	0.0	0	0.0	0	0.0	0	0.0	0	0.0	0	0.0
		Skill mix - Multidisciplinary teams	1	14.3	0	0.0	0	0.0	3	14.3	0	0.0	0	0.0	0	0.0	0	0.0	0	0.0	0	0.0	0	0.0	0	0.0
		Skill mix – Communication & case discussion between distant health professionals	0	0.0	1	7.1	0	0.0	0	0.0	0	0.0	0	0.0	0	0.0	0	0.0	0	0.0	0	0.0	0	0.0	0	0.0
		Staff - Support	0	0.0	0	0.0	5	16.7	0	0.0	0	0.0	0	0.0	0	0.0	0	0.0	0	0.0	2	50.0	0	0.0	5	38.5
		Staff - Workload/work flow/intensity	0	0.0	0	0.0	0	0.0	1	4.8	0	0.0	0	0.0	2	33.3	1	20.0	0	0.0	0	0.0	0	0.0	0	0.0
		Staff - Continuity of care	0	0.0	0	0.0	0	0.0	0	0.0	0	0.0	0	0.0	0	0.0	0	0.0	0	0.0	0	0.0	0	0.0	0	0.0
		Staff/self – Shared decision-making	0	0.0	1	7.1	0	0.0	0	0.0	0	0.0	0	0.0	0	0.0	0	0.0	0	0.0	0	0.0	0	0.0	0	0.0
		Self – Self-management	0	0.0	0	0.0	0	0.0	0	0.0	0	0.0	0	0.0	0	0.0	0	0.0	0	0.0	0	0.0	0	0.0	0	0.0
		**Total By whom care is provided**	7	9.3	14	8.2	30	14.4	21	12.9	3	3.3	14	9.9	6	8.3	5	5.8	5	2.7	4	4.9	8	5.6	13	18.3

			Bahrain		Egypt		Jordan		Lebanon		Libya		Morocco		Oman		Palestine		Sudan		Syria		Tunisia		Yemen	
			N	%	N	%	N	%	N	%	N	%	N	%	N	%	N	%	N	%	N	%	N	%	N	%
	Where care is provided	Site of service delivery	0	0.0	0	0.0	1	33.3	0	0.0	0	0.0	0	0.0	0	0.0	0	0.0	2	18.2	2	22.2	1	5.9	2	28.6
		Physical structure, facilities & equipment	0	0.0	2	8.7	1	33.3	0	0.0	0	0.0	0	0.0	0	0.0	0	0.0	1	9.1	4	44.4	1	5.9	4	57.1
		Organizational scale	0	0.0	0	0.0	0	0.0	0	0.0	0	0.0	0	0.0	0	0.0	0	0.0	0	0.0	0	0.0	0	0.0	0	0.0
		Integration of services	0	0.0	0	0.0	0	0.0	0	0.0	0	0.0	0	0.0	0	0.0	0	0.0	0	0.0	0	0.0	0	0.0	0	0.0
		Continuity of care	0	0.0	0	0.0	0	0.0	0	0.0	0	0.0	0	0.0	0	0.0	0	0.0	1	9.1	0	0.0	0	0.0	0	0.0
		Primary healthcare	9	90.0	13	56.5	1	33.3	4	57.1	1	100	0	0.0	3	75.0	3	100	6	54.5	2	22.2	10	58.8	1	14.3
		Acute Care Settings	1	10.0	8	34.8	0	0.0	3	42.9	0	0.0	11	100	0	0.0	0	0.0	1	9.1	1	11.1	5	29.4	0	0.0
		Long Term Care	0	0.0	0	0.0	0	0.0	0	0.0	0	0.0	0	0.0	1	25.0	0	0.0	0	0.0	0	0.0	0	0.0	0	0.0
		Home care	0	0.0	0	0.0	0	0.0	0	0.0	0	0.0	0	0.0	0	0.0	0	0.0	0	0.0	0	0.0	0	0.0	0	0.0
		**Total Where care is provided**	10	13.3	23	13.5	3	1.4	7	4.3	1	1.1	11	7.7	4	5.6	3	3.5	11	5.9	9	11.0	17	12.0	7	9.9
	With what information & communication technology (ICT) is care provided	Health record systems	0	0.0	1	9.1	1	4.3	1	11.1	0	0.0	1	5.9	3	33.3	1	14.3	1	6.7	0	0.0	1	12.5	0	0.0
		Electronic health record	0	0.0	0	0.0	1	4.3	1	11.1	0	0.0	0	0.0	1	11.1	0	0.0	0	0.0	1	20.0	0	0.0	0	0.0
		Other ICT that support individuals who provide care	0	0.0	4	36.4	6	26.1	1	11.1	0	0.0	3	17.6	0	0.0	1	14.3	0	0.0	1	20.0	2	25.0	0	0.0
		ICT that support individuals who receive care	0	0.0	0	0.0	2	8.7	0	0.0	2	20.0	0	0.0	0	0.0	0	0.0	0	0.0	0	0.0	0	0.0	0	0.0
		Diagnostic/medical equipment	4	66.7	0	0.0	1	4.3	2	22.2	8	80.0	1	5.9	1	11.1	1	14.3	12	80.0	0	0.0	3	37.5	0	0.0
		Information technology	0	0.0	3	27.3	0	0.0	1	11.1	0	0.0	2	11.8	1	11.1	1	14.3	0	0.0	1	20.0	1	12.5	0	0.0
		Electronic health record	0	0.0	0	0.0	0	0.0	0	0.0	0	0.0	0	0.0	0	0.0	0	0.0	0	0.0	0	0.0	0	0.0	0	0.0
		Technology assessment	1	16.7	0	0.0	1	4.3	3	33.3	0	0.0	0	0.0	1	11.1	0	0.0	0	0.0	1	20.0	0	0.0	0	0.0
		Innovation & research	1	16.7	3	27.3	11	47.8	0	0.0	0	0.0	10	58.8	2	22.2	3	42.9	2	13.3	1	20.0	1	12.5	0	0.0
		**Total With what information and communication technology (ICT) is care provided**	4	5.3	11	6.4	23	11.0	9	5.5	10	11.1	17	12.0	9	12.5	7	8.1	15	8.1	5	6.1	8	5.6	0	0.0
	With what level of	Quality monitoring	1	33.3	9	50.0	1	33.3	5	83.3	4	100	6	30.0	4	50.0	0	0.0	11	64.7	2	33.3	11	55.0	2	25.0

			Bahrain		Egypt		Jordan		Lebanon		Libya		Morocco		Oman		Palestine		Sudan		Syria		Tunisia		Yemen	
			N	%	N	%	N	%	N	%	N	%	N	%	N	%	N	%	N	%	N	%	N	%	N	%
	quality & safety is care provided	systems																								
		Safety monitoring systems	0	0.0	5	27.8	0	0.0	0	0.0	0	0.0	8	40.0	0	0.0	1	33.3	1	5.9	0	0.0	2	10.0	0	0.0
		Patient safety	0	0.0	3	16.7	1	33.3	0	0.0	0	0.0	2	10.0	0	0.0	0	0.0	0	0.0	0	0.0	1	5.0	0	0.0
		Quality	1	33.3	1	5.6	1	33.3	0	0.0	0	0.0	4	20.0	4	50.0	0	0.0	0	0.0	1	16.7	2	10.0	1	12.5
		Regulatory Interventions	1	33.3	0	0.0	0	0.0	1	16.7	0	0.0	0	0.0	0	0.0	2	66.7	5	29.4	3	50.0	4	20.0	5	62.5
		**Total With what level of quality and safety is care provided**	3	4.0	18	10.5	3	1.4	6	3.7	4	4.4	20	14.1	8	11.1	3	3.5	17	9.1	6	7.3	20	14.1	8	11.3
		**Total Delivery Arrangements**	33	44.0	81	47.4	140	67.0	112	68.7	89	98.9	114	80.3	67	93.1	32	37.2	105	56.5	66	80.5	101	71.1	74	104.2
**Implementation strategies**	Consumer-targeted strategies	Information or education provision	0	0.0	23	51.1	26	72.2	21	77.8	5	62.5	15	78.9	10	71.4	10	83.3	19	61.3	6	60.0	11	78.6	6	60.0
		Behaviour change support	0	0.0	17	37.8	9	25.0	6	22.2	3	37.5	2	10.5	2	14.3	1	8.3	10	32.3	2	20.0	3	21.4	3	30.0
		Skills and competencies development	0	0.0	2	4.4	1	2.8	0	0.0	0	0.0	0	0.0	0	0.0	1	8.3	1	3.2	1	10.0	0	0.0	0	0.0
		(Personal) Support	0	0.0	0	0.0	0	0.0	0	0.0	0	0.0	0	0.0	1	7.1	0	0.0	1	3.2	0	0.0	0	0.0	0	0.0
		Communication and decision-making facilitation	0	0.0	2	4.4	0	0.0	0	0.0	0	0.0	2	10.5	0	0.0	0	0.0	0	0.0	1	10.0	0	0.0	0	0.0
		System participation	0	0.0	1	2.2	0	0.0	0	0.0	0	0.0	0	0.0	1	7.1	0	0.0	0	0.0	0	0.0	0	0.0	1	10.0
		**Total Consumer Targeted Strategies**	0	0.0	45	26.3	36	17.2	27	16.6	8	8.9	19	13.4	14	19.4	12	14.0	31	16.7	10	12.2	14	9.9	10	14.1
	Provider-targeted strategies	Educational materials	0	0.0	7	63.6	23	82.1	7	70.0	1	50.0	5	45.5	6	75.0	5	41.7	13	40.6	3	33.3	7	87.5	4	66.7
		Educational meetings	0	0.0	2	18.2	4	14.3	2	20.0	1	50.0	2	18.2	0	0.0	0	0.0	4	12.5	2	22.2	1	12.5	2	33.3
		Educational outreach visits	0	0.0	1	9.1	0	0.0	0	0.0	0	0.0	0	0.0	0	0.0	2	16.7	0	0.0	0	0.0	0	0.0	0	0.0
		Local opinion leaders	0	0.0	0	0.0	0	0.0	0	0.0	0	0.0	0	0.0	0	0.0	0	0.0	0	0.0	0	0.0	0	0.0	0	0.0
		Local consensus processes	0	0.0	0	0.0	0	0.0	0	0.0	0	0.0	0	0.0	0	0.0	0	0.0	0	0.0	0	0.0	0	0.0	0	0.0
		Peer review	0	0.0	0	0.0	0	0.0	0	0.0	0	0.0	0	0.0	0	0.0	0	0.0	0	0.0	0	0.0	0	0.0	0	0.0
		Audit and feedback	0	0.0	0	0.0	0	0.0	1	10.0	0	0.0	2	18.2	2	25.0	0	0.0	7	21.9	0	0.0	0	0.0	0	0.0
		Reminders and prompts	0	0.0	0	0.0	0	0.0	0	0.0	0	0.0	0	0.0	0	0.0	0	0.0	0	0.0	0	0.0	0	0.0	0	0.0
		Tailored interventions	0	0.0	1	9.1	0	0.0	0	0.0	0	0.0	1	9.1	0	0.0	4	33.3	3	9.4	2	22.2	0	0.0	0	0.0

			Bahrain		Egypt		Jordan		Lebanon		Libya		Morocco		Oman		Palestine		Sudan		Syria		Tunisia		Yemen	
			**N**	**%**	**N**	**%**	**N**	**%**	**N**	**%**	**N**	**%**	**N**	**%**	**N**	**%**	**N**	**%**	**N**	**%**	**N**	**%**	**N**	**%**	**N**	**%**
		Patient-mediated interventions	0	0.0	0	0.0	1	3.6	0	0.0	0	0.0	1	9.1	0	0.0	0	0.0	0	0.0	0	0.0	0	0.0	0	0.0
		Multi-faceted interventions	0	0.0	0	0.0	0	0.0	0	0.0	0	0.0	0	0.0	0	0.0	1	8.3	5	15.6	2	22.2	0	0.0	0	0.0
		**Total Provider Targeted Strategies**	0	0.0	11	6.4	28	13.4	10	6.1	2	2.2	11	7.7	8	11.1	12	14.0	32	17.2	9	11.0	8	5.6	6	8.5
		**Total Implementation Strategies**	0	0.0	56	32.7	64	30.6	37	22.7	10	11.1	30	21.1	22	30.6	24	27.9	63	33.9	19	23.2	22	15.5	16	22.5
**Non-relevant study but with ''Health System and Policy' implications**			35	46.7	86	50.3	39	18.7	51	31.3	19	21.1	39	27.5	10	13.9	36	41.9	49	26.3	31	37.8	47	33.1	11	15.5
